# Evaluation of integrated neuromuscular training on the recovery of joint injury: A protocol for systematic review and meta-analysis

**DOI:** 10.1097/MD.0000000000028737

**Published:** 2022-02-04

**Authors:** Jing Zeng, Qing Liu, Zhengfang Lei, Zhe Sun, Yang Wang

**Affiliations:** Chengdu Sport University, Chengdu, Sichuan Province, China.

**Keywords:** integrated neuromuscular training, joint injury, meta-analysis, restoration, system evaluation

## Abstract

**Background:**

Integrated neuromuscular training (INT) is a comprehensive and holistic training method. It combines general functional movement training with specialized strength, balance, speed, sensitivity, coordination, enhanced training or rapid telescopic compound training. From the existing research results, the mechanism of INT mainly lies in improving the proprioception of the human body and cognitive level to achieve the impact on the motor sensory system, so as to effectively prevent joint injury and promote the recovery after joint injury.

**Method:**

This article is assisted by the third and fourth authors to search the relevant literature. The search strategy is divided into 2 parts: English literature and Chinese literature. English literature search: the keywords “integrated neurological training”, “joint investigation”, and “restoration” are jointly searched with “meta analysis” and “system evaluation”, respectively. The search databases include PsycINFO, Science Direct, PubMed, Eric, and Willey. Chinese literature search: the keywords “integrated neuromuscular training”, “joint injury”, and “recovery” are jointly searched with “meta analysis” and “system evaluation”, respectively. The search databases include the general library of online publishing of academic journals of China Knowledge Network (CNKI) and the full-text database of excellent doctoral theses of China Knowledge Network (CNKI). At the same time, the supplementary search is carried out through literature backtracking, Google Scholar.

**Results:**

This study will provide new evidence for the effect of INT on the recovery of joint injury.

**Conclusion:**

To provide a method to help the prevention and restoration of joint injury by INT.

**INPLASY registration number:**

INPLASY2021120136.

## 1 Introduction

Integrated neuromuscular training (INT) is a comprehensive and holistic training method. It combines general functional movement training with specialized strength, balance, speed, sensitivity, coordination, enhanced training or rapid telescopic compound training. The human body can effectively prevent human joint injury and promote the recovery effect after injury through INT effect.^[1–4]^ From the existing research results, the action mechanism of INT is mainly through the impact of INT on the motor sensory system, through the integration of multisensory information to the domination of the central nervous system, and finally through the motor output of skeletal muscle, so as to realize local sensation, neuromuscular control, posture control. Improve the ability of sports performance and sports injury prevention, so as to achieve the impact on the motor sensory system. Based on this, it is becoming more and more important to carry out INT to prevent joint injury and recover after joint injury. In the existing research results, neuromuscular training includes super isometric movement training, core strength and balance training, resistance strength training, intermittent speed training, and sensitivity training.^[3,5–7]^

However, there are still some disputes about the training effect and training mechanism of INT. There are still many differences between the conclusions of the research on the influence of INT training on sprint ability, sensitivity, and balance ability. The existing meta-analysis only focuses on the treatment and recovery of anterior cruciate ligament (ACL) injury by INT, the research on other joints of lower limbs is relatively weak, and there is almost no relevant research on upper limbs and spinal column joints. In addition, the impact of INT cycle, intervention frequency and intervention time on joint injury, the differences of intervention effects among different populations, ages, and genders, and whether different test methods and methods can accurately evaluate and reflect the recovery of injured populations are also worth studying. Based on this, this study uses the meta-analysis method to systematically analyze the randomized controlled trial (RCT) tests related to the influence of INT on joint injury at home and abroad, and test the recovery effect of INT on joint injury, in order to provide reference for the practice of the recovery effect of INT on different joint injuries.

## 2 Methods

### 2.1 Registration

This study protocol systematic review has been registered in INPLASY. The registration number is INPLASY2021120136.

### 2.2 Inclusion criteria

#### 2.2.1 Study designs

We will study the effect of INT on joint injury. The study will be selected according to the following criteria:

(1) **Study type:** RCT.

#### 2.2.2 Participants

The people who were confirmed to have joint injury.

#### 2.2.3 Intervention measures

The experimental group received medical treatments and INT intervention scheme after medical treatments. The control group only received the medical treatments.

#### 2.2.4 Outcome measures

**Primary outcomes:** Test the range of motion of the patient's joints.

**Secondary outcomes:** Test the patient's sensitivity, physical stability, speed and strength of muscle completion.

#### 2.2.5 Exclusion criteria

Review and comment research or non-Chinese and English literature; in the study, only the experimental group, no control group or the control group is the literature of blank control; literature published in the form of abstracts, research that cannot obtain the full text, or literature with incomplete research data and unsuccessful contact with the author.

### 2.3 Data sources

According to the participant intervention control outcome study design (PICOS) principle, the third and fourth authors of this paper searched PsycINFO, Science Direct, PubMed, Eric, Willey, China Knowledge Network (CNKI) Academic Journal Online Publishing General Library, and China Knowledge Network (CNKI) excellent doctoral thesis full-text database by computer to collect relevant research on the impact of INT on joint injury repair. The time limit of injury retrieval is from the establishment of the database to December 2021.

### 2.4 Data collection and analysis

#### 2.4.1 Search strategy

In addition, the references of the retrieved literature are traced to supplement the relevant literature. The retrieval adopts the combination of subject words and free words. The keywords are “integrated neuromuscular training”, “joint injury”, and “recovery”, etc; taking CNKI search library as an example. The specific search strategy of this study is shown in Box 1. The same strategies are used in other electronic databases.

Box 1 CNKI searching

1. #1 Integrated neuromuscular training
2. #2 Joint injury
3. #3 Knee joint injury
4. #4 Hip joint injury
5. #5 Ankle joint injury
6. #6 Shoulder joint injury
7. #7 Elbow joint injury
8. #8 Wrist joints injury
9. #9 Thoracic vertebra injury
10. #10 Lumbar vertebra injury
11. #11 Cervical vertebra injury

#### 2.4.2 Study selection

Literature screening includes 4 stages: retrieval, preliminary screening, confirmation and inclusion. Two reviewers (the third and fourth authors of this paper) separately screened the literature according to the inclusion and exclusion criteria, and divided the literature into “inclusion”, “uncertainty”, and “exclusion”. If there are differences, they shall be discussed together or settled jointly with a third party (the first author of this paper). In the 3 stages of preliminary screening, confirmation, and inclusion, the “kappa values” of evaluator consistency were 0.64, 0.75, and 0.92, respectively. According to the criterion of “kappa” value between 0.40 and 0.59, the consistency is considered good, between 0.60 and 0.74, and between 0.75 and above, the consistency is considered very good.^[8]^

#### 2.4.3 Measures of effect

Meta-analysis was performed using Review Manager 5.4. The evaluation criteria of effect quantity are: 0.2 is small effect quantity, 0.5 is medium equivalent stress quantity, and 0.8 is large effect quantity.^[9]^ Q-test and statistics were used to test the heterogeneity between studies. Q value significantly indicates heterogeneity between studies; describe the proportion of the variance between studies in the overall variance. The judgment criteria are: 25% is low heterogeneity, 50% is medium heterogeneity, and 75% is high heterogeneity.^[10]^

$$d=\frac{M_{1}-M_{2}}{sd_{pooled}},\;sd_{pooled}=\sqrt{\frac{(n_{1}-1){s_{1}}^{2}+(n_{2}-1){S_{2}}^{2}}{n_{1}+n_{2}-2}}$$

In social science research, heterogeneous group effects generally exist, and researchers recommend the random effect model.^[11]^ In addition, Borenstein et al^[12]^ believe that the selection of the model depends on judging in advance whether there are the same real effects between studies and the purpose of analysis.

#### 2.4.4 Data extraction

**Figure 1:**
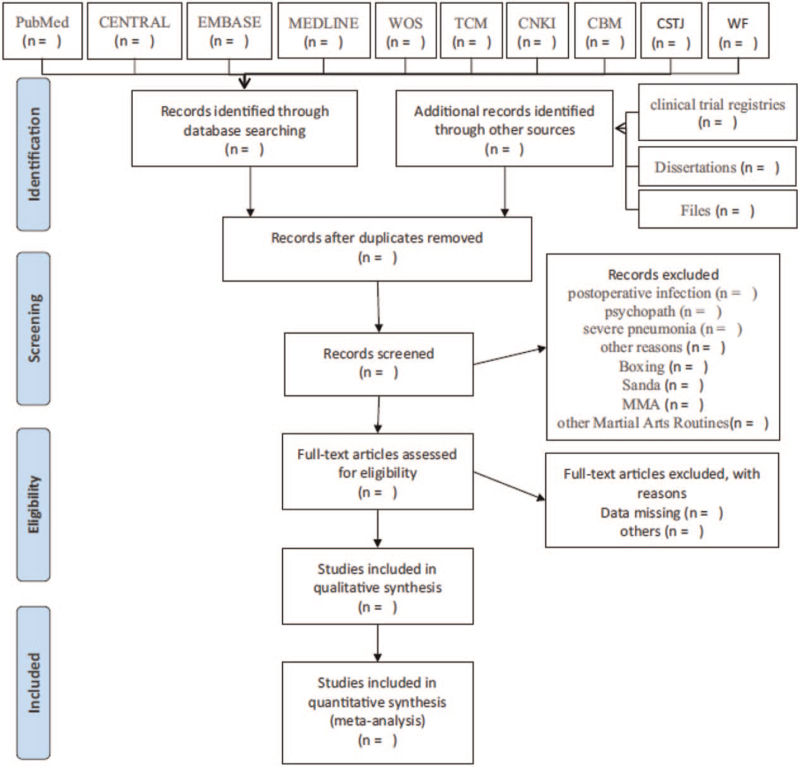
Flow chart of the study.

Researchers independently searched the literature, discussed with the first author, and screened, confirmed, and included the literature according to the criteria of inclusion and exclusion. In the first screening of literature, the main method is to exclude studies that are obviously irrelevant to the research topic by reading the title, and then clearly understand whether the article is RCT by further reading the abstract and full text; whether the data reported in the literature is complete. If the original data cannot be obtained, contact the author (Fig. 1). At the same time, extract the data such as “first author”, “publication time”, “intervention mode”, and “utility”; extract the key elements of bias risk assessment; extract the result indicators and data (Table 1).

**Table 1:** Basic characteristics of included literature.

				Intervening methods			Outcome of indicators	
Included reserches	Number of cases (T/C)	Training history (T/C)	Average age (T/C)	T	C	Intervening time (wk)	PO	SO
							Range of joint motion	Sensitivity; physical stability; speed and strength of muscle completion.

C = control group, PO = primary outcomes, SO = secondary outcomes, T = test group.

#### 2.4.5 Risk of bias assessment

Publication bias means that the research results with statistical significance are easier to publish, while the research results without statistical significance are often rejected, which makes it difficult to collect the literature without statistical significance in the meta-analysis process, resulting in systematic errors between the included research and the actual research, and then affect the meta-analysis results. In order to ensure the quality and effectiveness of the included literature, the bias risk of the included literature was comprehensively evaluated according to the evaluation tool for the bias risk of RCTs in the Cochrane system evaluator manual. The study used Review Manager 5.3 software to evaluate the methodological quality of the sources of bias (selective bias, implementation bias, measurement bias, loss of follow-up bias, and other bias) included in the literature.

The horizontal axis of funnel chart is the standardized mean difference (effect quantity), and the vertical axis is its standard error. If the effect quantity of funnel diagram is evenly and symmetrically distributed on the left and right, it indicates that meta-analysis has no publication bias, on the contrary, it has publication bias.^[13]^ Rosenthal's loss of safety factor (NFS), Egger linear regression test, and shear compensation method were further used to test publication bias. First, if NFS is less than the critical value 5K + 10 (K refers to the number of independent effects included in the meta-analysis), it indicates that there may be publication deviation^[13]^ (Table 2).

**Table 2:** Overall effect quantity and heterogeneity test results.

k	n	d	95%CI	Q	df	I^2^ (%)
						

CI = confidence interval, d = Cohen d, k = number of independent effects, n = sample size. ^∗^*P* < .05, ^∗∗^*P* < .01, ^∗∗∗^*P* < .001 indicates significant effect amount. Q = significant heterogeneity within the group (the same below).

#### 2.4.6 Dealing with missing data

Dealing with missing data. In order to ensure the integrity and accuracy of the article data, when the data are lost, we will contact the corresponding author to obtain complete data. If complete data cannot be obtained through this method, we will delete incomplete data for the sake of data integrity and accuracy.

#### 2.4.7 Subgroup analysis

The random effect model was used for subgroup analysis to investigate the role of 3 regulatory variables: treatment mode, treatment time, and disease cycle in the recovery effect of INT on joint injury.

#### 2.4.8 Sensitivity analysis

Sensitivity analysis was used to analyze the research quality, intervention methods, publication types, etc. If the heterogeneity is large, the method of eliminating the literature 1 by 1 should be used for sensitivity analysis.

#### 2.4.9 Grading the quality of evidence

The risk of bias included in the study was independently evaluated by 2 researchers through the Cochrane manual 5.1.0 bias risk assessment tool.^[14]^

## 3 Discussion

This study mainly introduces how to systematically analyze the prevention and recovery of joint injury from INT in the aspects of literature download process, screening and inclusion criteria, data collection and analysis, heterogeneity analysis, sensitivity analysis, and subgroup analysis. Previous studies focused more on the recovery of ACL, while this study will discuss the recovery effect of joints with high probability of human injury, which is not limited to the recovery effect of ACL. However, the article has some limitations, such as no analysis of non-English literature, and the sample size of literature screening needs to be further expanded.

### 3.1 Limitations

Due to joint injury and recovery is a global problem, we should pay attention to non-English language analysis in order to ensure the accuracy of the conclusion, so that other scholars can further study recovery effect of sports medicine on joint injury.

## Author contributions

**Conceptualization:** Jing Zeng.

**Data curation:** Zhengfang Lei, Zhe Sun.

**Formal analysis:** Jing Zeng.

**Methodology:** Qing Liu.

**Software:** Yang Wang.

**Writing – original draft:** Jing Zeng.

**Writing – review & editing:** Qing Liu.
